# Respiratory variations of inferior vena cava fail to predict fluid responsiveness in mechanically ventilated patients with isolated left ventricular dysfunction

**DOI:** 10.1186/s13613-019-0589-5

**Published:** 2019-10-07

**Authors:** Hongmin Zhang, Qing Zhang, Xiukai Chen, Xiaoting Wang, Dawei Liu, Chinese Critical Ultrasound Study Group (CCUSG)

**Affiliations:** 1 Department of Critical Care Medicine, Peking Union Medical College Hospital, Chinese Academy of Medical Sciences and Peking Union Medical College, Beijing, China; 2 Pittsburgh Heart, Lung, Blood and Vascular Institute, University of Pittsburgh, School of Medicine, Pittsburgh, PA USA; 3 Department of Critical Care Medicine, Peking Union Medical College Hospital, Chinese Academy of Medical Sciences, 1# Shuai Fu Yuan, Dong Cheng District, Beijing, 100730 China

**Keywords:** Echocardiography, Inferior vena cava, Fluid responsiveness, Heart function, Critically ill

## Abstract

**Background:**

Respiratory variation of inferior vena cava is problematic in predicting fluid responsiveness in patients with right ventricular dysfunction. However, its effectiveness in patients with isolated left ventricular systolic dysfunction (ILVD) has not been reported. We aimed to explore whether inferior vena cava diameter distensibility index (dIVC) can predict fluid responsiveness in mechanically ventilated ILVD patients.

**Methods:**

Patients admitted to the intensive care unit who were on controlled mechanical ventilation and in need of a fluid responsiveness assessment were screened for enrolment. Several echocardiographic parameters, including dIVC, tricuspid annular plane systolic excursion (TAPSE), left ventricular ejection fraction (LVEF), and LV outflow tract velocity–time integral (VTI) before and after passive leg raising (PLR) were collected. Patients with LV systolic dysfunction only (TAPSE ≥ 16 mm, LVEF < 50%) were considered to have isolated left ventricular systolic dysfunction (ILVD).

**Results:**

One hundred and twenty-nine subjects were enrolled in this study, among them, 28 were labelled ILVD patients, and the remaining 101 were patients with normal LV function (NLVF). The value of dIVC in ILVD patients was as high as that in NLVF patients, (20% vs. 16%, *p* = 0.211). The ILVD group contained a much lower proportion of PLR responders than NLVF patients did (17.9% vs. 53.2%, *p *< 0.001). No correlation was detected between dIVC and ΔVTI in ILVD patients (*r* = 0.196, *p *= 0.309). dIVC was correlated with ΔVTI in NLVF patients (*r* = 0.722, *p *< 0.001), and the correlation was strengthened compared with that derived from all patients (*p *= 0.020). A receiver-operating characteristic (ROC) analysis showed that the area-under-the-curve (AUC) of dIVC for determining fluid responsiveness from ILVD patients was not statistically significant (*p *= 0.251). In NLVF patients, ROC analysis revealed an AUC of 0.918 (95% CI 0.858–0.978; *p *< 0.001), which was higher than the AUC derived from all patients (*p *= 0.033). Patients with LVEF below 40% had a lower ΔVTI and fewer PLR responders than those with LVEF 40–50% and LVEF above 50% (*p *< 0.001).

**Conclusion:**

dIVC should be used with caution when critically ill patients on controlled mechanical ventilation display normal right ventricular function in combination with abnormal left ventricular systolic function.

## Background

Only 50% of haemodynamically unstable critically ill patients are fluid responders, and volume overload is detrimental to nonresponders; therefore, fluid responsiveness is frequently assessed in daily practice in the intensive care unit (ICU) [1, 2]. The diameter of the inferior vena cava (IVC) and the variation in this quantity are determined by the circulating blood volume and right ventricular (RV) function. For ICU patients on controlled mechanical ventilation, the IVC distensibility index (dIVC) was shown by previous studies to be a reliable indicator of fluid responsiveness [3, 4]. In contrast, some researchers have drawn different conclusions, and even the results of several meta-analyses about dIVC have been in-homogeneous [5, 6].

IVC diameters vary with the intra-abdominal pressure change during the respiratory cycle. In a number of clinical contexts, dIVC may not accurately predict fluid responsiveness. Prior studies have identified a relatively low tidal volume or a high PEEP as reasons for the inaccuracy of measuring fluid responsiveness from the IVC [7]. Another important factor is abdominal hypertension. Elevated intra-abdominal pressure reduces IVC size regardless of volume status, and is likely to affect the reliability of IVC-based fluid responsiveness assessment [8, 9].

RV dysfunction, including chronic pulmonary hypertension or RV myocardial infarction or severe tricuspid regurgitation, leads to significantly increased IVC size and reduced variation. Therefore, in the presence of RV dysfunction, the fluid responsiveness also cannot be reflected from IVC [10, 11]. By contrast, if there is isolated left ventricular systolic function (ILVD), i.e., the RV function remains normal and the left ventricular (LV) systolic function is impaired, can IVC variation still be reflective of fluid responsiveness? ILVD is not a rare phenomenon in critically ill and is deemed an important mechanism of pulmonary oedema [12]. To the best of our knowledge, whether ILVD could influence the accuracy of dIVC has not been investigated. We hypothesized that when RV function is normal, while LV systolic function is impaired for various reasons, the IVC variation might not reflect fluid responsiveness. We thus performed this study to explore whether ILVD would affect the effectiveness of dIVC in assessing fluid responsiveness in mechanically ventilated critically ill patients.

## Patients and methods

### Study population

Patients admitted to the Peking Union Medical College Hospital ICU from 1 July 2018 to 1 January 2019 were screened for enrolment within the first 24 h after admission.

The inclusion criteria were as follows: mechanical ventilation without spontaneous breathing effort and the need for an assessment of fluid responsiveness due to hypotension, tachycardia, oliguria, and hyperlactatemia (with the decision to assess fluid responsiveness made at the discretion of an attending physician).

Patients with the following conditions were excluded from the study: RV dilatation and paradoxical septal motion, or tricuspid annular plane systolic excursion (TAPSE) below 16 mm [13, 14]; rhythm characteristic of atrial fibrillation; valvular diseases such as severe mitral, aortic or tricuspid stenosis or regurgitation; left ventricular outflow tract (LVOT) obstruction that was diagnosed by a high-velocity, late-peaking, dagger-shaped continuous-wave Doppler signal in conjunction with a peak gradient of at least 30 mmHg [15]; intra-abdominal pressure equal or above 12 mmHg [16]; an inadequate echocardiographic image for measurement; contraindication to passive leg raising (PLR) including hip or spine surgery, intracranial hypertension and intra-aortic balloon pump support; and absence of an echocardiography examiner. We also excluded post-cardiac surgery patients because of lower IVC acquisition rate resulted from subcostal drainage and modified cardiac structure which prevented precise assessment of necessary parameters.

The study was conducted in compliance with the Declaration of Helsinki and was approved by the ethics committee of our institution. Informed consent was obtained from the next of kin.

### Echocardiography

Echocardiograms were recorded within the first 24 h of ICU admission using an echocardiograph (CX50, PHILIPS, USA) with a 2.5-MHz phased-array probe. Images were saved for offline analysis. Two intensivists who were experienced in echocardiography performed the echo examination. Electrocardiograms were recorded continuously during the echo examination. Three cardiac cycles were analyzed and averaged. M-mode and Doppler echocardiographic measurements were taken according to standard protocols.

The left ventricular ejection fraction (LVEF) was obtained using a modified biplane Simpson’s method from apical two- and four-chamber views. The mitral annular plane systolic excursion (MAPSE) was obtained from the apical 4-chamber view by positioning the cursor along the lateral mitral ring and measuring the difference between the highest and lowest point of the M-mode sinusoid wave [17]. TAPSE was also obtained from the apical 4-chamber view by positioning the M-mode cursor along the lateral part of the tricuspid valve ring [18]. The LVOT velocity–time integral (VTI) was obtained from pulsed Doppler imaging by positioning the sample volume at the LVOT approximately 0.5 cm below the aortic valve [19]. The diameter of the IVC was measured in M-mode through the subcostal longitudinal plane, just upstream of the origin of the suprahepatic vein. The patients were all in controlled ventilation and the diameter of IVC was measured using M-mode. dIVC was calculated as (maximum diameter on inspiration − minimum diameter on expiration)/minimum diameter on expiration [3].

### Passive leg raising manoeuvre

PLR is considered a reliable method of predicting volume responsiveness, even in spontaneously breathing patients [20, 21]. In a recent meta-analysis, the area-under-the-curve (AUC) of PLR for predicting fluid responsiveness in patients with shock could be up to 0.95 [22]. The PLR manoeuvre was performed by first placing the patient in a semi-recumbent position with the head elevated at 45° and then positioning the patient supine with the legs straight and elevated at 45° for 2 min. The VTI was measured before PLR and after 90 s of PLR. We managed to obtain an optimal VTI spectrum within 120 s [23].

### Other parameters collected

Demographic information, Acute Physiology and Chronic Health Evaluation (APACHE) II scores, Sequential Organ Failure Assessment (SOFA) scores, reasons for admission, currently used vasoactive agents and ICU mortality were collected for all patients. We also recorded each patient’s heart rate (HR), mean arterial pressure (MAP) and ventilator settings at the time of the echo examination.

### Definition

RV dysfunction was defined as TAPSE below 16 mm [13]. LV dysfunction was defined as LVEF below 50%, as in prior studies [24, 25]. ILVD patients were defined as those with only LV systolic dysfunction (TAPSE ≥ 16 mm, LVEF < 50%). Fluid responsiveness was defined as a 10% increase in VTI after PLR [9, 21, 26].

### Statistical analysis

Statistical analysis was performed using the statistical software package SPSS 13.0 (SPSS, Inc., Chicago, Illinois, USA). Continuous data were expressed as the mean ± SD or the median and the interquartile range. Categorical variables were presented as frequency and percentages. The distributions of the continuous values were assessed for normality by the Kolmogorov–Smirnov test. Group comparisons were performed by Student’s *t* test, the Mann–Whitney *U* test, the Chi-squared test, or Fisher’s exact test where appropriate. Spearman’s correlation coefficients and their corresponding *p* values were calculated to assess the variable relationships. Receiver-operating characteristic (ROC) curves were generated and the AUCs were then calculated and compared for all patients, patients with normal left ventricular function (NLVF) and ILVD groups. Intraobserver and interobserver variabilities in TAPSE, dIVC, LVEF, and VTI were assessed in 20 randomly selected patients and were tested using both paired *t* tests and intraclass correlation coefficients (ICCs). An ICC > 0.8 was considered excellent agreement. All *p* values were two tailed and were considered significant for *p *< 0.05.

## Results

### Measurement variability

The intraobserver variabilities in TAPSE, dIVC, LVEF, and VTI were minimal. The interobserver variability analysis revealed that ICCs for TAPSE, dIVC, LVEF, and VTI were: 0.967 (95% CI 0.918–0.987), 0.940 (95% CI 0.850–0.976), 0.900 (95% CI 0.748–0.960), and 0.953 (95% CI 0.877–0.982), respectively.

### General characteristics of all patients

**Fig. 1:**
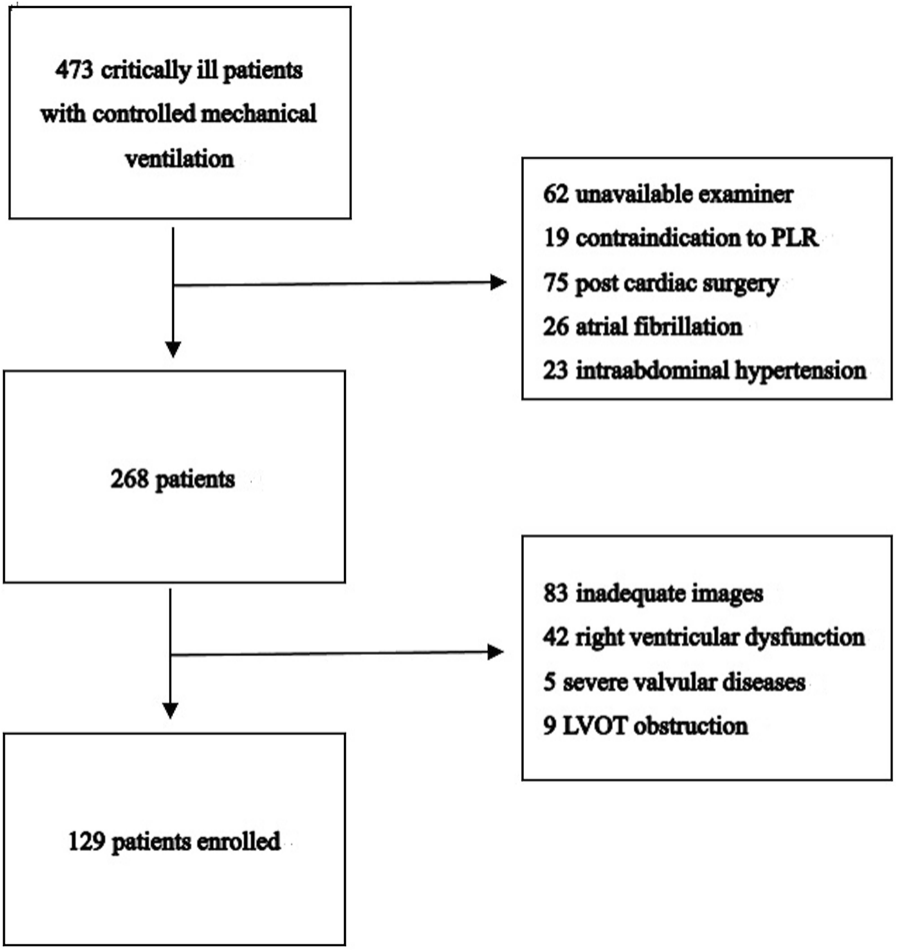
Flow chart of the study. *PLR* passive leg raising, *LVOT* left ventricular outflow tract

A total of 473 patients were screened for enrolment. Three hundred and forty-four patients were excluded because of unavailability of an examiner, diagnoses that could be confounding factors, contraindication to PLR and inadequate images (Fig. 1). One hundred and twenty-nine patients were enrolled in this study, and the general characteristics are illustrated in Table 1. The mean age of the subjects was 59 years, and 57.4% were men. The mean APACHE II and SOFA scores were 16 ± 6 and 6 ± 4, respectively. The reasons for admission included noncardiac surgery (47.2%), various types of shock (38.0%), respiratory failure (10.1%), and other reasons (4.7%), i.e., diabetic ketoacidosis, cerebral disease, and kidney failure. The median echo examination timing was at 10 h after ICU admission. The median volume administered before echo was 1595 ml. The ICU mortality was 12.4%.

**Table 1:** General characteristics

Categories	Findings (*n* = 129)
Age (year)	59 ± 19
Sex (male, %)	74 (57.4%)
APACHEII	16 ± 6
SOFA	6 ± 4
Reason for admission (*n*, %)	
Noncardiac surgery	61 (47.2%)
Circulatory shock	49 (38.0%)
Respiratory failure	13 (10.1%)
Others^a^	6 (4.7%)
Type of shock (*n*, %)	
Septic shock	28 (21.7%)
Cardiogenic shock	11 (8.5%)
Haemorrhagic shock	5 (3.9%)
Other types	5 (3.9%)
Tidal Volume (ml/kg)	6.7 (6.3, 7.3)
PEEP (cmH_2_O)	5.5 ± 1.7
NE infusion (*n*, %)	55 (42.6%)
NE dose (μg/kg/min)	0.2 (0.1, 0.35)
Lactate (mmol/L)	2.7 (1.9, 3.5)
Timing of echo (hour from admission)	10 (5, 20)
Volume administered before examination (ml)	1595 (800, 2462)
Prognosis	
ICU mortality (*n*, %)	16 (12.4%)

*APACHE* acute physiology and chronic health evaluation, *SOFA* sequential organ failure assessment, *PEEP* positive end-expiratory pressure, *NE* norepinephrine, *CVP* central venous pressure, *ICU* intensive care unit

^a^Diabetic ketoacidosis, cerebral disease and kidney failure

### Haemodynamic and echocardiographic parameters of the patients

According to the echocardiographic results, 28 patients displayed ILVD and the remaining 101 patients who displayed normal biventricular functions were labelled as NLVF patients. The HR and MAP were similar in these two groups of patients. The TASPE in the two groups were not significant different (20.8 ± 3.4 mm vs. 22.1 ± 3.7 mm, *p *= 0.111). The MAPSE and LVEF in ILVD patients were lower (11.9 ± 3.8 mm vs. 14.9 ± 3.3 mm, *p *< 0.001 and 38% vs. 65%, *p *< 0.001). The ILVD patients had lower baseline VTI (*p *= 0.025). The two groups had similar end-expiratory IVC diameters (14.9 ± 3.4 mm vs. 15.8 ± 3.8 cm, *p *= 0.278).

**Fig. 2:**
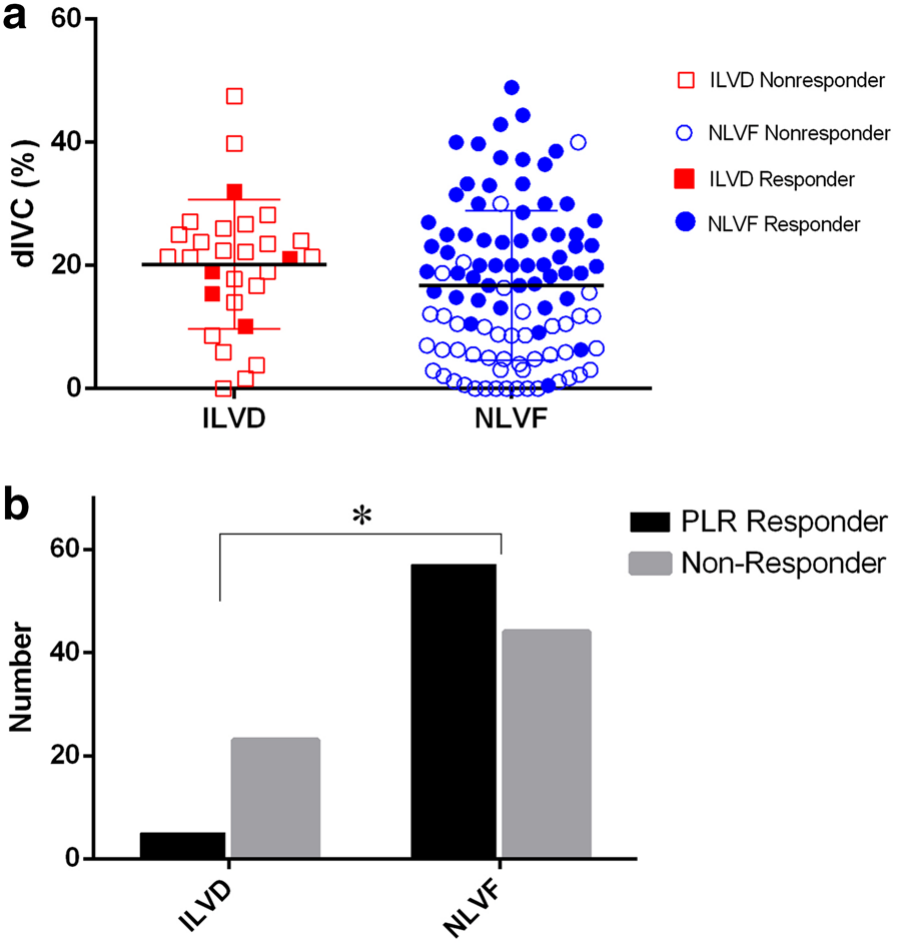
Distribution of dIVC and proportion of PLR responders in ILVD and NLVF patients. **a** There was no difference in dIVC value between ILVD and NLVF patients, *p *= 0.211. **b** ILVD patients had much lower proportion of PLR responders than that in NLVF patients, *p *< 0.001. *ILVD* isolated left ventricular systolic dysfunction, *NLVF* normal left ventricular function, *dIVC* inferior vena cava distensibility index, *PLR* passive leg raising

The ILVD patients had dIVC value as high as that in NLVF patients (20% vs. 16%, *p *= 0.211), while the ILVD group had a much lower proportion of PLR responders than the NLVF group (17.9% vs. 53.2%, *p *< 0.001) (Table 2, Fig. 2a, b).

**Table 2:** Hemodynamic and echocardiographic parameters of the patients

Categories	ILVD (*n* = 28)	NLVF (*n* = 101)	*p*
HR (bpm)	91 ± 18	88 ± 19	0.325
MAP (mmHg)	78 ± 13	79 ± 15	0.695
MAPSE (mm)	11.3 ± 2.8	14.9 ± 3.3	< 0.001
TAPSE (mm)	20.8 ± 3.4	22.1 ± 3.7	0.111
LVEF (%)	38 (29, 45)	65 (60, 71)	< 0.001
IVCEE (mm)	14.9 ± 3.4	15.8 ± 3.8	0.278
dIVC (%)	20 (13, 24)	16 (6, 25)	0.211
VTI (cm)	17.7 ± 5.1	20.1 ± 4.9	0.025
VTI post PLR (cm)	18.0 ± 5.0	21.6 ± 4.8	0.003
Number of PLR responders (*n*, %)	5 (17.9%)	57 (56.4%)	< 0.001

*ILVD* isolated left ventricular systolic dysfunction, *NLVF* normal left ventricular function, *HR* heart rate, *MAP* mean arterial pressure, *VTI* velocity–time integral, *PLR* passive leg raising, *IVC EE* diameter of inferior vena cava at end expiration, *MAPSE* mitral annular plane systolic excursion, *TAPSE* tricuspid annular plane systolic excursion, *LVEF* left ventricle ejection fraction, *dIVC* inferior vena cava distensibility index, *MRLF* mismatch of right and left heart function, *PLR* passive leg raising

### Correlation analysis of dIVC and ΔVTI

**Fig. 3:**
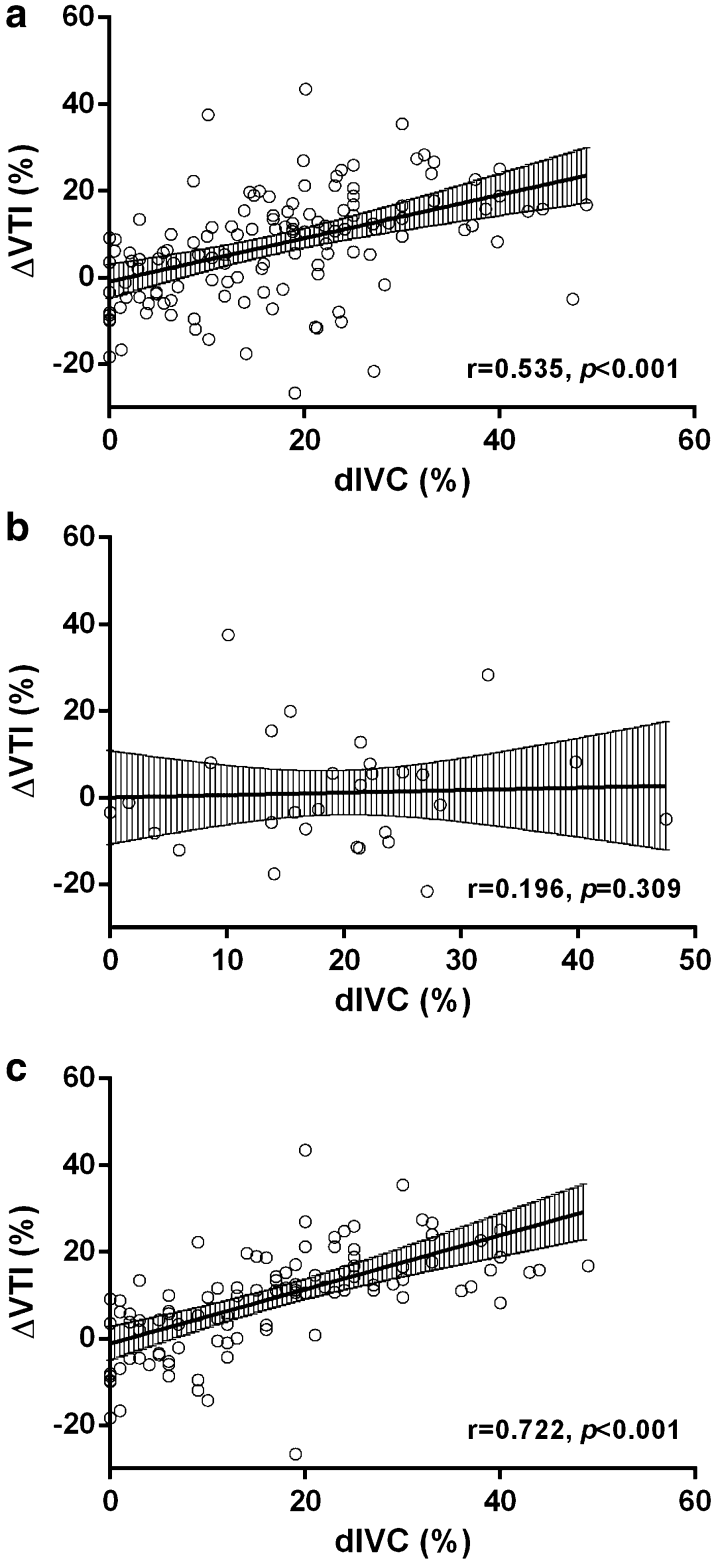
Correlation of dIVC and △VTI. **a** Correlation of dIVC and △VTI in all patients, *r* = 0.535, *p *< 0.001. **b** Correlation of dIVC and △VTI in ILVD patients, *r* = 0.196, *p *= 0.309. **c** Correlation of dIVC and △VTI in NLVF patients, *r* = 0.722, *p *< 0.001. Correlation of dIVC and △VTI derived from NLVF patients was strengthened than that derived from all patients, *p *= 0.020. *ILVD* isolated left ventricular systolic dysfunction, *NLVF* normal left ventricular function; *dIVC* inferior vena cava distensibility index, △*VTI* velocity–time integral change after passive leg raising

dIVC was associated with ΔVTI among all patients, *r* = 0.535, *p *< 0.001. No correlation between those two variables was found in ILVD patients, *r* = 0.196, *p *= 0.309. When we specifically examined NLVF patients, dIVC was still associated with ΔVTI in that group, *r* = 0.722, *p *< 0.001, and the correlation was strengthened, *Z* = − 2.336, *p *= 0.020 (Fig. 3a–c).

### ROC analysis of dIVC for the detection of fluid responsiveness

**Fig. 4:**
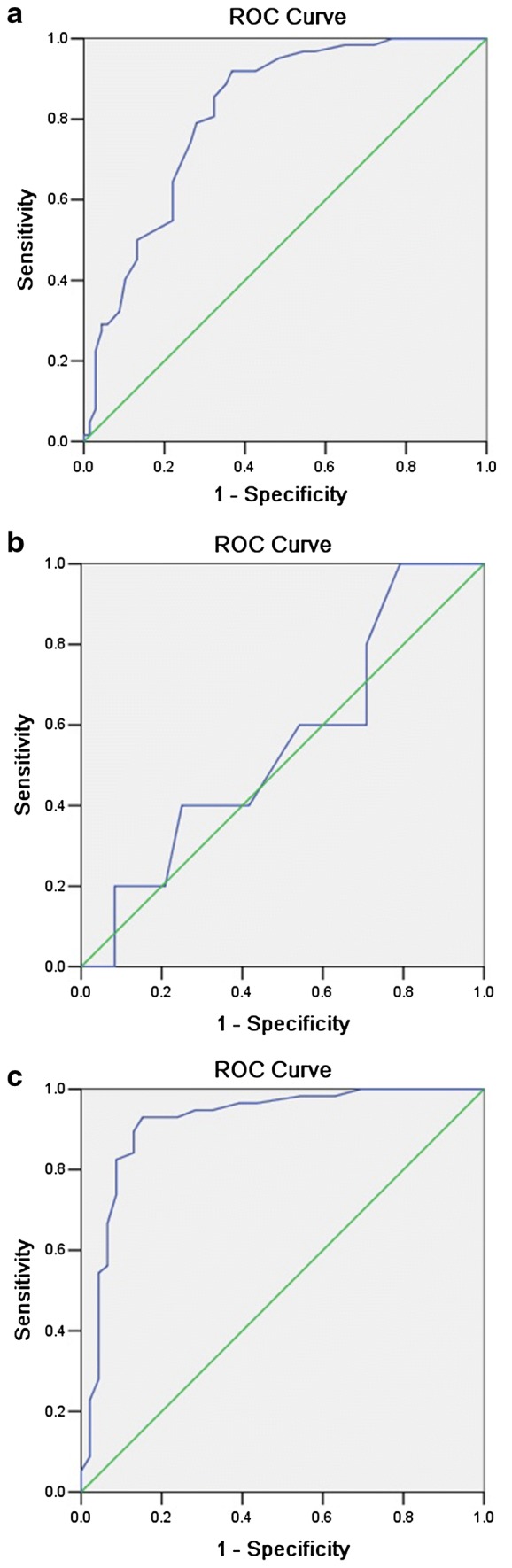
ROC analysis of dIVC for the detection of fluid responsiveness. **a** Area-under-the-curve (AUC) of dIVC for the detection of fluid responsiveness in all patients 0.815 (95% CI 0.742–0.889; *p *< 0.001). **b** In ILVD patients, the AUC was only 0.550 (95% CI 0.283–0.817; *p *= 0.729). **c** In NLVF patients, the ROC analysis revealed an AUC of 0.918 (95% CI 0.858–0.978; *p *< 0.001), which was statistically significant compared with the AUC derived from all patients, *p *= 0.033. *ILVD* isolated left ventricular systolic dysfunction, *NLVF* normal left ventricular function; *dIVC* inferior vena cava distensibility index

To evaluate the sensitivity and specificity of dIVC for assessing fluid responsiveness, we generated ROC curves. The ROC analysis showed that the AUC of dIVC for determining fluid responsiveness was 0.815 in all patients (95% CI 0.742–0.889; *p *< 0.001). In patients with ILVD, the AUC of dIVC for determining fluid responsiveness was 0.550 (95% CI 0.283–0.817; *p *= 0.729). After the ILVD patients were excluded, i.e., in NLVF group, the ROC analysis revealed an AUC of 0.918 (95% CI 0.858–0.978; *p *< 0.001), which was significantly different from the AUC derived from all patients, *Z* = 2.134, *p *= 0.033 (Table 3, Fig. 4a–c).

**Table 3:** dIVC for the detecting of fluid responsiveness

Categories	AUC	95% CI	*p*	Optimum cutoff (%)	Sen (%)	Spe (%)	PPV (%)	NPV (%)
All patients (*n* = 129)	0.815	0.742–0.889	< 0.001	16.5	79.0	72.1	72.4	78.7
NLVF (*n* = 101)^a^	0.918	0.858–0.978	< 0.001	14.5	82.7	87.0	89.7	84.5
ILVD (*n* = 28)	0.550	0.283–0.817	0.729	21.5	40.0	58.3	17.3	81.7

*NLVF* normal left ventricular function, *ILVD* isolated left ventricular systolic dysfunction, *dIVC* inferior vena cava distensibility index, *AUC* area-under-the-curve, *CI* confidence interval, *Sen* sensitivity, *Spe* specificity, *PPV* positive predictive value, *NPV* negative predictive value

^a^In comparison with AUC derived from all patients, *p *= 0.033

### Analysis of patients with different LVEF levels

We divided all the patients into those with LVEF below 40%, LVEF 40–50%, and LVEF above 50%. dIVC was not statistically different among patients with different LVEF levels (*p *= 0.247). Patients with LVEF below 40% had the lowest ΔVTI and fewest patients with PLR response (*p *< 0.001) (Table 4).

**Table 4:** dIVC and ΔVTI in patients with different LVEF

Categories	LVEF > 50% (*n* = 101)	LVEF 40–50% (*n* = 11)	LVEF < 40% (*n* = 17)	*p*
dIVC (%)	16 (6, 25)	19 (15, 22)	20 (12, 26)	0.247
ΔVTI (%)	10.8 (2.0, 16.6)	5.5 (−1.3, 18.1)	−2.9 (−9.8, 3.3)	< 0.001
Number of PLR responders (*n*, %)	57 (56.4%)	4 (36.4%)	1 (5.9%)	< 0.001

*dIVC* inferior vena cava distensibility index, *VTI* velocity–time integral, *LVEF* left ventricle ejection fraction, *PLR* passive leg raising

## Discussion

In this study, we investigated the use of dIVC for fluid responsiveness assessment in critically ill patients on controlled mechanical ventilation. We demonstrated that when there was an isolated LV systolic dysfunction, i.e., normal RV function in combination with impaired LV function, the effectiveness of dIVC for predicting fluid responsiveness was compromised.

We opted to use PLR to assess fluid responsiveness, because fluid challenge provides a risk of volume overload in a high proportion of critically ill patients. The effectiveness of PLR has been validated by other researchers [27]. The differences in the cutoff value could be due to different samples, different criteria for stroke volume increase, or even the use of different equations to calculate dIVC [3, 4]. We chose TAPSE to represent RV function on the basis that TAPSE is easy to measure, less operator dependent and is correlated with the biplane Simpson RV EF and myocardial performance index [28].

The present study showed that ILVD is not uncommon in critically ill patients. After excluding patients with RV dysfunction, patients with normal RV function and LV dysfunction still accounted for 21% of our study sample. Chockalingam et al. identified the common causes of acute left ventricular dysfunction in critically ill patients, including acute coronary syndrome (ACS), takotsubo cardiomyopathy, and global hypokinesis induced by sepsis or other insults [29]. The aforementioned diagnosis frequently results in abnormal LVEF, but rather not necessarily result in concomitant RV dysfunction [30–33]. Thus, ILVD is not rare in ICU. Although we were unable to collect enough information about the patients’ LV function status before ICU admission, some patients might have preexisting LV dysfunction. In addition, we believe that some acute conditions also account for this phenomenon, such as newly onset of stressed cardiomyopathy or septic cardiomyopathy. Pulido et al. performed echo examinations of 68 severe sepsis and septic shock patients. The frequency of LV systolic dysfunction was 42.6% and only approximately half of the patients had RV dysfunction simultaneously [33].

This study revealed that there was no correlation between dIVC and ΔVTI in patients with ILVD. We speculated that it was the mismatch of RV and LV function that led to this result. Normally, the output of the right and left ventricle is the same and a patient would respond to fluid loading only when both ventricles operate on the ascending portion of the Frank–Starling curve. If one of the ventricles operates on the flat portion of the curve, the cardiac output will not increase significantly in response to volume expansion [35]. The notion that IVC variation could reflect fluid responsiveness is also based on the theory that the two ventricles usually have equal function. If the left ventricle is impaired first, the right ventricle usually becomes involved as well, which is because the underlying injury may affect both ventricles, or the two ventricles may affect each other through ventricular interdependence. However, in ILVD patients, the function of the two ventricles might become incompatible and respond differently to preload increase, and high dIVC might merely reflect a potential stroke volume increase in the RV and not the LV [12, 34]. Therefore, the mismatch between the biventricular functions in ILVD patients could diminish the accuracy of volume status assessment of IVC. In this study, we excluded patients with RV dysfunction, because IVC was not able to accurately reflect volume status in this situation. For patients with RV dysfunction such as chronic pulmonary hypertension, RV infarction or severe tricuspid regurgitation, fluid responsiveness may still exist, while the IVC diameter is large and its variation is small [11]. However, when the RV and LV function are both impaired, whether IVC can reflect fluid responsiveness need further investigation.

We found that the ILVD patients had higher dIVC than NLVF patients, though not statistically different. IVC reflects the interaction of venous return and RV function. The physicians had various ways to understand the patients’ heart function including medical history, physical examination, or even cardiac ultrasound. They might choose more conservative strategy in terms of volume administration in patients with LV dysfunction.

Fluid responsiveness is one of the key steps in haemodynamic management [36]. The echocardiographic plane of the IVC is easily obtained at the bedside and is less dependent on image quality than other echocardiographic imaging parameters. With an increasing number of ICU physicians being trained in focused cardiac ultrasound examination, IVC variation are used increasingly often in the management of critically ill patients [37–39]. Nonetheless, for ILVD patients, if fluid was administered according to the value of dIVC, the chance of a stroke volume increase would very slim. Fluid therapy guided solely by dIVC would put the patient at risk of occurrence or exacerbation of pulmonary oedema. Therefore, ILVD should be taken into account when the IVC is used to predict fluid responsiveness. In addition to assessing IVC variation before administering fluid, one should simultaneously examine heart function, which could improve clinical decision making and lower the risk of volume overload.

We also assessed the difference of patients with mildly depressed LV function and those with markedly low LVEF. The results showed that the latter group was less likely to respond to volume expansion. Rather than diagnosing patients as LV dysfunction, the exact LVEF value should be taken into consideration when treating ILVD patients. Our results were in line with a recent study focusing on hemodynamic type of septic shock [40]. They discovered that septic shock patients with LV systolic dysfunction had a median LVEF of 29%. Therefore, although LVEF below 50% was the cutoff value for LV dysfunction, a lower LVEF seemed to be more clinically relevant.

## Limitations

This study has several limitations. First, this study was conducted at a single centre, and the enrolled population was heterogeneous. We were unable to provide the exact aetiologies for ILVD, which can result from ACS or coronary arterial disease, takotsubo cardiomyopathy, septic cardiomyopathy, or other causes. However, the enrolment heterogeneity and lack of an aetiology would not prevent the deduction of the conclusion, which is based on the echocardiographic appraisal of heart function. Second, although the patients with intra-abdominal hypertension before the PLR test were excluded, we did not know the real value of intra-abdominal pressure during PLR. As elevated intra-abdominal pressure can impede PLR-induced venous return, it might result in false negatives of this manoeuvre [41]. Third, we chose to use LVEF, an afterload-dependent parameter, as the marker of LV dysfunction. Other parameters such as tissue Doppler or strain would be more appropriate than LVEF for reflecting the intrinsic contractility of the left ventricle. However, those markers are more machine- or operator dependent than LVEF. Finally, we did not include patients with biventricular dysfunction in this study, and we failed to differentiate patients with LV diastolic dysfunction from patients with a normal LVEF. The above-mentioned diagnoses also could be confounding factors affecting the utility of IVC effectiveness for fluid responsiveness assessment, and future studies are warranted.

## Conclusions

Mismatch between RV and LV function should be taken into consideration in assessing the fluid responsiveness of ICU patients through IVC variation. dIVC should be used with caution when critically ill patients on controlled mechanical ventilation display normal right heart function in combination with abnormal left heart systolic function.

## Data Availability

All data sets used and analyzed during the current study are available from the corresponding author on reasonable request.

## Abbreviations

ILVD: isolated left ventricular systolic function
NLVF: normal left ventricular function
IVC: inferior vena cava
dIVC: IVC distensibility index
TAPSE: tricuspid annular plane systolic excursion
LVEF: left ventricular ejection fraction
VTI: velocity–time integral
ICU: intensive care unit
RV: right ventricle
ACP: acute cor pulmonale
LVOT: left ventricular outflow tract
MAPSE: mitral annular plane systolic excursion
PLR: passive leg raising
APACHE: acute physiology and chronic health evaluation
SOFA: sequential organ failure assessment
HR: heart rate
MAP: mean arterial pressure
CVP: central venous pressure
ROC: receiver-operating characteristic
ICC: intraclass correlation coefficient
PEEP: positive end-expiratory pressure
AUC: area-under-the-curve
PPV: positive predictive value
NPV: negative predictive value
ACS: acute coronary syndrome
AMI: acute myocardial infarction
